# Giant Magnetocaloric Effect in a Honeycomb Spiral Spin‐Liquid Candidate

**DOI:** 10.1002/advs.202510086

**Published:** 2025-08-26

**Authors:** Yuqian Zhao, Xun Chen, Zongtang Wan, Zhaohua Ma, Yuesheng Li

**Affiliations:** ^1^ Wuhan National High Magnetic Field Center and School of Physics Huazhong University of Science and Technology Wuhan 430074 China

**Keywords:** adiabatic demagnetization refrigeration, frustrated honeycomb‐lattice antiferromagnet, magnetocaloric effect, spiral spin liquid

## Abstract

Unlike conventional magnetic states, which lack degeneracy, the spiral spin liquid (SSL) fluctuates among degenerate spiral configurations, with ground‐state wave vectors forming a continuous contour or surface in reciprocal space. At low temperatures, the field‐induced crossover from the polarized ferromagnetic state to the SSL results in a large entropy increase and decalescence, indicating its potential for magnetic cooling. However, magnetic cooling using a SSL has yet to be reported. Here, the magnetocaloric effect and cooling performance of single‐crystal GdZnPO, a spin‐7/2 honeycomb‐lattice SSL candidate, are investigated under a magnetic field *H* < *H*_c_ (*µ*_0_ *H*_c_ ∼ 12 T) applied perpendicular to the honeycomb plane and below the crossover temperature (∼2 K). For *H* ⩾ *H*_c_, GdZnPO enters a polarized non‐degenerate ferromagnetic state. These results demonstrate that GdZnPO exhibits a giant low‐temperature magnetocaloric effect near *H*_c_, surpassing other magnetocaloric materials. This giant magnetocaloric effect is well‐explained by the frustrated honeycomb spin model of GdZnPO, suggesting the stability of the SSL below *H*_c_ down to very low temperatures. Additionally, its magnetic cooling performance remains robust up to at least 4.5 K, making GdZnPO a promising candidate for magnetic refrigeration down to ∼36 mK through cycling the applied magnetic field within a narrow range.

## Graphical abstract

The frustrated honeycomb‐lattice single‐crystal GdZnPO, a spiral spin‐liquid (SSL) candidate, exhibits a giant magnetocaloric effect (with a large Grüneisen parameter *Γ*_m_ = 4.4 T^−1^) and exceptional cooling performance near the crossover magnetic field (*H*_c_), surpassing other known magnetocaloric materials. These findings underscore GdZnPO's promise for magnetic cooling and open avenues for further research into SSL candidates for similar applications.

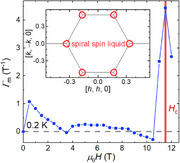

## 1 Introduction

Paramagnetic materials with a large magnetocaloric effect (MCE) were initially proposed as a means to achieve temperatures well below 1 K, and have since developed into an effective low‐temperature refrigeration technique.^[^ ^1^, ^2^ ^]^ Compared to conventional ^3^He‐based refrigeration techniques (such as ^3^He‐^4^He dilution refrigerators), magnetic cooling is more cost‐efficient, compact, and easier to handle,^[^ ^3^, ^4^, ^5^, ^6^, ^7^ ^]^ as ^3^He gas is scarce, non‐renewable, and expensive.^[^ ^8^ ^]^ In the pursuit of higher MCE and improved efficiency in magnetic cooling, a novel approach based on a field‐induced critical point (CP) in strongly correlated and frustrated spin systems has been proposed and successfully tested.^[^ ^9^, ^10^, ^11^, ^12^ ^]^ This technique offers at least three key advantages: (1) Strongly correlated magnets typically have a larger volumetric entropy capacity, *S*_V_ = *R*ln(2*s*+1)/*V*_m_, compared to highly diluted paramagnetic salts, enabling further reduction in magnet size and ensuring good cooling power. Here, *V*_m_ is the volume per mole of spins and *s* is the spin quantum number. (2) The MCE of strongly correlated magnets can be significantly enhanced, with a low‐temperature magnetic Grüneisen parameter *Γ*_m_ = (d*T*/d*H*)/(*µ*_0_ *T*) ∼ 0.6–3.8 T^−1^,^[^ ^10^, ^11^, ^12^, ^13^ ^]^ as the applied magnetic field approaches the critical value. (3) During the refrigeration cycles, the magnetic field change can be confined to a relatively narrow range, where *Γ*_m_ is large, reducing eddy current effects in metal parts and minimizing energy cost.

**Figure 1:**
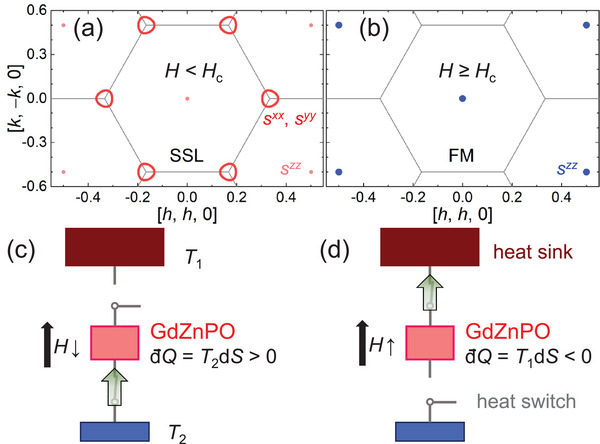
Magnetic cooling using GdZnPO. a,b) Ground‐state wave vectors (**Q**_G_) of the spiral spin liquid (SSL) and the polarized ferromagnetic (FM) phase, with magnetic fields applied along the *c* axis, for *H* < *H*_c_ and *H* ⩾ *H*_c_, respectively. The in‐plane spin structure factor *s* ^*xx*^ or *s* ^*yy*^ forms a degenerate **Q**_G_ contour around the K{1/3, 1/3} point in the SSL, while the out‐of‐plane component *s* ^*zz*^ is nonzero only at the Γ{0, 0} point for *H* ≠ 0.^[^ ^24^ ^]^ *H*_c_ is the crossover field, and the gray lines represent Brillouin zone boundaries. c,d) Schematic diagrams of heat absorption (dQ > 0) and release (dQ < 0) processes due to entropy changes.

A spiral spin liquid (SSL) in a frustrated spin system fluctuates cooperatively among degenerate spiral configurations, with ground‐state wave vectors (**Q**_G_) forming a continuous contour or surface in reciprocal space, depending on the system.^[^ ^14^, ^15^, ^16^ ^]^ The low‐energy topological excitations and defects in a SSL, such as spin vortices, antivortices,^[^ ^17^, ^18^ ^]^ and momentum vortices,^[^ ^16^, ^19^ ^]^ may be used in small‐scale antiferromagnetic spintronic devices,^[^ ^20^, ^21^, ^22^ ^]^ offering topologically protected memory and logic operations^[^ ^23^ ^]^ without magnetic field leakage. Because of the degenerate spiral contour (see **Figure** **1**a) or surface, the specific heat of a SSL (*C*_SSL_) remains large even at extremely low temperatures, *C*_SSL_(*T* → 0) → *C*_0_ (∼*R*/2 for a 2D classical SSL),^[^ ^14^, ^15^, ^24^ ^]^ in contrast to a conventional magnetic ordered phase (CMOP, see Figure 1b), where *C*_CMOP_(*T* → 0) → 0. As a result, the entropy (*S*) of the SSL is significantly higher than that of the conventional magnetic ordered phase at low temperatures. The crossover from the conventional magnetic ordered phase to the SSL in a frustrated spin system produces large decalescence, *Q* = $\int_{\mathrm{CMOP}}^{\mathrm{SSL}}TdS$, highlighting the potential of SSL candidates for magnetic cooling near the CP. However, to the best of our knowledge, magnetic cooling using a SSL has not yet been explored.

Under a magnetic field applied perpendicular to the plane, SSLs can exist over a wide range of interaction parameters in the easy‐plane (*D* ⩾ 0) frustrated honeycomb‐lattice model: $H=J_{1}\sum_{\left\langle j0,j1\right\rangle }s_{j0}\cdot s_{j1}+J_{2}\sum_{\left\langle \langle j0,j2\rangle \right\rangle }s_{j0}\cdot s_{j2}+D\sum_{j0}{\left(s_{j0}^{z}\right)}^{2}-\mu_{0}Hg\mu_{B}\sum_{j0}s_{j0}^{z}$, where *J*_1_ and *J*_2_ are the first‐ and second‐nearest‐neighbor Heisenberg couplings, respectively, and *µ*_B_ is Bohr magneton.^[^ ^25^, ^26^, ^27^, ^28^, ^29^, ^30^ ^]^ In this model, **Q**_G_ forms a continuous contour around the Γ{0, 0} point (1/2 > |*J*_2_/*J*_1_| > 1/6) or the K{1/3, 1/3} point (|*J*_2_/*J*_1_| > 1/2) in reciprocal space. In the previous work,^[^ ^24^ ^]^ we identified the structurally disorder‐free rare‐earth Gd^3+^ (*s* = 7/2) antiferromagnet GdZnPO^[^ ^31^, ^32^ ^]^ as a candidate for this prototypical honeycomb‐lattice model, with *J*_1_ ∼ −0.39 K, *J*_2_ ∼ 0.57 K, *D* ∼ 0.30 K, and the *g* factor *g* ∼ 2. With |*J*_2_/*J*_1_| ∼ 1.5 (>1/2), GdZnPO undergoes a crossover to a unique SSL with a spiral contour around the K point below T* ∼ 2 K, from a thermally paramagnetic phase above T*.^[^ ^24^ ^]^ The SSL contour is analytically given by **Q**_G_ = *h*_G_ **b**_1_+*k*_G_ **b**_2_ with the condition $\cos\left(2\pi h_{G}\right)+\cos\left(2\pi k_{G}\right)+\cos\left(2\pi h_{G}+2\pi k_{G}\right)=\left[J_{1}^{2}/\left(4J_{2}^{2}\right)-3\right]/2$ (Figure 1a), which is independent of *H* below the crossover value *H*_c_. Here, **b**_1_ and **b**_2_ are the reciprocal lattice vectors.

Below T*, the GdZnPO spin system stays in the putative SSL with the degenerate contour unchanged for *H* < *H*_c_, but crosses over to a substantially polarized ferromagnetic (FM) phase when *H* ⩾ *H*_c_.^[^ ^24^ ^]^ The crossover magnetic field is analytically given by *µ*_0_ *H*_c_ = $s\left[2D+3J_{1}+9J_{2}+J_{1}^{2}/\left(4J_{2}\right)\right]/\left(g\mu_{B}\right)$ (∼12 T). Because of the spatial localization of the 4*f* Gd^3+^ electrons, both *J*_1_ and *J*_2_ interactions are relatively weak, making the crossover field (*µ*_0_ *H*_c_ ∼ 12 T) experimentally accessible. Moreover, single‐crystal samples are available, enabling the magnetic field to be applied perpendicular to the honeycomb plane, i.e., along the *c* axis. Therefore, single‐crystal GdZnPO is an ideal candidate for magnetic cooling near *H*_c_.

In this work, we investigate the low‐temperature MCE of single‐crystal GdZnPO with the magnetic field applied along the *c* axis to assess its magnetic cooling capacity. GdZnPO, with a large *S*_V_ = 0.424 JK^−1^cm^−3^, exhibits a giant MCE with a normalized Grüneisen ratio *Γ* ^norm^ = *µ*_0_ *HΓ*_m_ = 28–51 between 11 and 12 T (near the crossover field *µ*_0_ *H*_c_ ∼ 12 T) at low temperatures, where *Γ*_m_ = 2.5–4.4 T^−1^ is the magnetic Grüneisen parameter. Both *Γ* ^norm^ and magnetic cooling performance remain high up to at least 4.5 K (>T*), suggesting the promising potential of GdZnPO, a honeycomb‐lattice SSL candidate, for adiabatic demagnetization refrigeration. These results may encourage further research into the use of other SSL candidates^[^ ^14^, ^18^, ^22^, ^33^, ^34^, ^35^, ^36^, ^37^, ^38^, ^39^ ^]^ for magnetic cooling.

## 2 Results

### 2.1 Magnetocaloric Effect

**Figure 2:**
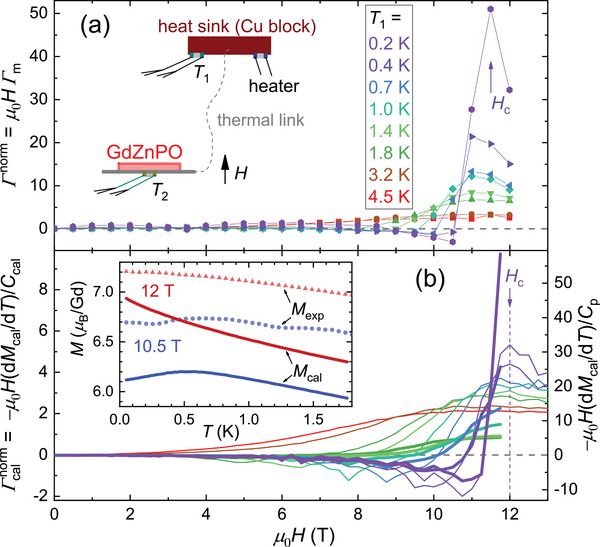
Magnetocaloric effect of GdZnPO. a) Magnetic field dependence of the normalized Grüneisen ratio, *Γ* ^norm^ = *µ*_0_ *HΓ*_m_, measured with an alternating‐field frequency of 0.005 Hz, where *Γ*_m_ is the magnetic Grüneisen parameter. Inset: Diagrammatic sketch of the experimental setup. b) Grüneisen ratio calculated from Monte Carlo simulations of magnetization (*M*_cal_) and specific heat (*C*_cal_), $\Gamma_{\mathrm{cal}}^{\mathrm{norm}}$ = −*µ*_0_ *H*(d*M*_cal_/d*T*)/*C*_cal_ (thin lines). Thick lines show −*µ*_0_ *H*(d*M*_cal_/d*T*)/*C*_p_, using experimental specific heat *C*_p_ instead. Inset: Temperature dependence of *M*_cal_ (lines) and experimental magnetization *M*_exp_ (points) at 10.5 and 12 T. The crossover field *H*_c_ is indicated.

The MCE characterizes the magnetic cooling performance of candidate compounds. In our measurements on GdZnPO, the alternating magnetic field is generated by the main superconducting magnet as *H*(*t*) = Δ*H*sin [2*π* *ν*(*t* − *t*_*H*_)] + *H* ^av^, which induces a sine wave signal for the sample temperature *T*_2_(*t*) ∼ Δ*T*sin [2*π* *ν*(*t* − *t*_*T*_)] + *T* ^av^, with *ν* = 0.005 Hz. The normalized Grüneisen ratio is given by *Γ* ^norm^(*T* = *T* ^av^ ∼ *T*_1_, *H* = *H* ^av^) = Δ*TH*/(*T*Δ*H*) (see **Figure** **2**a). The fits yield *t*_*T*_ ≈ *t*_*H*_ (Figure S1, Supporting Information), indicating good thermal contact between the GdZnPO crystals and the thermometer *T*_2_.^[^ ^40^ ^]^

**Figure 3:**
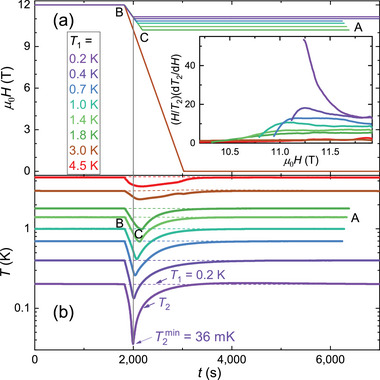
Quasi‐adiabatic demagnetization refrigeration using GdZnPO. a,b) Time dependence of the magnetic field and temperatures, *T*_1_ (dashed colored lines) and *T*_2_ (solid colored lines). The heat sink temperature, *T*_1_, was controlled at various values using the heater shown in the inset of Figure 2a. The inset of panel a) shows the corresponding Grüneisen ratio, (*H*/*T*_2_)(d*T*_2_/d*H*). A high field ramp rate, *v*_*H*_ = 0.6 Tmin^−1^, was used for *T*_1_ ⩾ 3 K, while *v*_*H*_ = 0.3 Tmin^−1^ was applied for *T*_1_ ⩽ 1.8 K to reduce eddy heat.

   Below 1.8 K, the maximum heat leak power due to the weak thermal link (see inset of Figure 2a) is calculated as $P_{L}^{\max}$ = *C*_tot_|Δ*T*|/*τ*, where the total specific heat *C*_tot_ is predominantly (≳99.8%) contributed by the magnetic specific heat of the GdZnPO sample, and *τ* = 400–600 s is the relaxation time (see C→A processes in **Figure** **3**b). At *T* ∼ 1.8 K, the specific heat of GdZnPO and its nonmagnetic reference YZnPO are 9.8 and 0.0156 JK^−1^mol^−1^, respectively.^[^ ^24^ ^]^ As *T* → 0 K, the lattice specific heat decreases following a *T* ^3^ behavior, much more rapidly than the magnetic contribution. In contrast, the maximum cooling or heating power due to the alternating field is $P_{C}^{\max}$ ∼ 2*π* *ν* *C*_tot_|Δ*T*|. Since *τ* ^−1^ ≪ 2*π* *ν*, it follows that $P_{L}^{\max}$ ≪ $P_{C}^{\max}$, confirming the quasi‐adiabatic conditions of our experiments. Moreover, doubling the frequency to *ν* = 0.01 Hz does not significantly increase *Γ*_m_ or *Γ* ^norm^ (Figure S2, Supporting Information), further validating our results.

The temperature and magnetic field dependence of the normalized Grüneisen ratio is simulated by Monte Carlo (MC) calculations via $\Gamma_{\mathrm{cal}}^{\mathrm{norm}}$ = −*µ*_0_ *H*(d*M*_cal_/d*T*)/*C*_cal_ (thin lines in Figure 2b).^[^ ^40^ ^]^ At low temperatures, $\Gamma_{\mathrm{cal}}^{\mathrm{norm}}$ peaks at the crossover field *µ*_0_ *H*_c_ ∼ 12 T of the many‐body model.^[^ ^24^ ^]^ Experimentally, *Γ* ^norm^ also peaks at ∼11.5 T (∼*µ*_0_ *H*_c_), where the magnetization *M* approaches its fully polarized value of *sg* ∼ 7 *µ*_B_/Gd, at low temperatures (Figure S2, Supporting Information). Therefore, the maximum field of our superconducting magnet, *µ*_0_ *H* = 12 T, is sufficient to substantially polarize the GdZnPO spin system. However, $\Gamma_{\mathrm{cal}}^{\mathrm{norm}}$ is smaller than the experimental *Γ* ^norm^, near *H*_c_. This discrepancy is primarily due to the spin quantization (*s* = 7/2) in the real GdZnPO compound. This quantization reduces the entropy per mole of spins to a finite value of *R*ln(2*s*+1), thereby strongly suppressing the specific heat at low temperatures. Replacing the overestimated specific heat from the classical model (*C*_cal_) with the experimentally measured value (*C*_p_) significantly reduces the discrepancy in the Grüneisen ratio, as shown by the thick lines in Figure 2b. The comparison between experimental and calculated specific heat was extensively discussed in our previous work on GdZnPO.^[^ ^24^ ^]^

Moreover, the classical MC calculations yield a crossover field of *µ*_0_ *H*_c_ = 12 T, which appears slightly higher than the experimental value. However, the low‐*T* d*M*/d*H* remains larger than the Van Vleck susceptibility ($\chi_{\mathrm{vv}}^{∥}$ ∼ 0.3 cm^3^mol^−1^) even at 12 T (Figure S3, Supporting Information), indicating that the GdZnPO spin system is not yet fully polarized at this field. This discrepancy is also likely due to quantum fluctuations associated with the finite spin quantum number *s* = 7/2 of the Gd^3+^ ions, which are not captured by the classical model.

The magnetocaloric effect can be quantitatively evaluated using magnetization (*M*) and specific heat (*C*_p_) measurements, via *Γ*_m_ = −(d*M*/d*T*)/*C*_p_. The low‐*T* *C*_m_ (∼*C*_p_) exhibits a sharp drop as the applied magnetic field approaches to the crossover (or critical) value of *µ*_0_ *H*_c_ ∼ 12 T (Figure S3, Supporting Information), which predominantly contributes to the observed giant low‐temperature magnetocaloric effect. The Van Vleck magnetization is expected to be temperature‐independent^[^ ^24^ ^]^ and can thus be safely neglected in simulations of the magnetocaloric effect. At low temperatures, a negative value of *Γ*_m_ implies that d*M*/d*T* > 0, meaning the magnetization *M* increases with temperature *T*. For example, at 0.2 K and 10.5 T, we observe *Γ*_m_ < 0 in both the experimental and simulated data (see Figure 2), and correspondingly, *M* increases with *T* (see the inset of Figure 2b). Negative *Γ*_m_ has also been widely reported at low temperatures in other frustrated magnets, such as *α*‐RuCl_3_^[^ ^41^ ^]^ and TmMgGaO_4_.^[^ ^13^ ^]^ In weakly correlated spin systems at finite temperatures, or strongly correlated ones at high temperatures, magnetization follows the Curie‐Weiss law under the mean‐field approximation, giving *Γ*_m_ > 0. Thus, observing *Γ*_m_ < 0 in a strongly correlated insulating magnet likely reflects complex many‐body spin correlations emerging at low temperatures (<|*θ*_w_|). Here, *θ*_w_ (∼−12 K for GdZnPO) is the Curie–Weiss temperature determined from magnetic susceptibility measurements.^[^ ^24^ ^]^

Below ∼T*, as *µ*_0_ *H* decreases to ∼10.5 T, *Γ* ^norm^ changes sign (Figure 2a), as confirmed by the raw alternating‐field data (Figure S1, Supporting Information). This feature is well reproduced by MC simulations, where *M*_cal_ increases at ∼12 T but decreases at ∼10.5 T as *T* approaches 0 K, aligning with the noisy experimental magnetization *M*_exp_. *M*_exp_ exceeds *M*_cal_ due to the Van Vleck contribution (inset of Figure 2b). As *T* → 0 K, the magnetic field where *Γ* ^norm^ = 0 approaches *H*_c_, where *Γ* ^norm^ reaches a maximum (see Figure 2), indicating the second‐order nature of the field‐induced crossover.^[^ ^41^, ^42^ ^]^ Additionally, the high‐resolution magnetization measured at 50 mK shows no evident magnetic hysteresis (Figure S2, Supporting Information), confirming that the field‐induced crossover is second‐order or higher. The directly determined Grüneisen parameter *Γ*_m_ is also roughly consistent with the noisy data obtained indirectly using experimentally measured *M* and *C*_p_, where *Γ*_m_ ∼ −(d*M*/d*T*)/*C*_p_ (Figure S4, Supporting Information).

As the GdZnPO spin system becomes thermally paramagnetic above T*,^[^ ^24^ ^]^ *Γ* ^norm^ remains positive and moderately high (∼3.3 at 4.5 K and 11.5 T) across the full range of ∼0 and 12 T (see Figure 2a and **Table** **1**). In contrast, we highlight that the observed *Γ* ^norm^ ∼ 51 (≫3.3) well below T* and near the crossover field is exceptionally high, arising from the crossover between the degenerate SSL and the non‐degenerate FM phases, which forms the core finding of this work. At *H* < *H*_c_ in the *J*_1_‐*J*_2_ frustrated honeycomb‐lattice model, the SSL is predicted to remain highly stable without “order by disorder” down to very low temperatures.^[^ ^30^ ^]^ This is confirmed by the giant magnetocaloric effect observed in this work and our previous thermodynamic measurements on GdZnPO,^[^ ^24^ ^]^ supporting its use in magnetic cooling down to the mK range (see below).

**Table 1:** Volumetric entropy capacity (*S*_V_ in JK^−1^cm^−3^), maximum Grüneisen parameter (*Γ*_m_ in T^−1^), normalized ratio (*Γ* ^norm^ = *µ*_0_ *HΓ*_m_), magnetic cooling efficiency (*Q*_2_/*Q*_1_), and low‐*T* operational field range (*µ*_0_ *H*_sta_−*µ*_0_ *H*_end_ in T) of GdZnPO, compared to other typical magnetocaloric materials.

compounds	*S*_V_	*Γ*_m_	*Γ* ^norm^	*Q*_2_/*Q*_1_	*µ*_0_ *H*_sta_−*µ*_0_ *H*_end_
**GdZnPO** (this work)	**0.424**	**4.44**	**51**	**0.60(2)**	**12−11.2**
Na_2_BaCo(PO_4_)_2_^[^ ^12^ ^]^	0.056	3.8	6.7	—	4−1.4
Cs_2_CuCl_4_^[^ ^45^ ^]^	0.043	3.4	29	0.57	10−8.5
NaYbP_2_O_7_^[^ ^46^ ^]^	0.064	3.2	0.9	—	5−0
Cu(NO_3_)_2_ ·2.5H_2_O^[^ ^47^ ^]^	0.059	2.7	12	—	5−2.9
KBaGd(BO_3_)_2_^[^ ^48^, ^49^, ^50^ ^]^	0.192	1.4‐3.1	0.8‐2.7	—	6−0.75
KBaYb(BO_3_)_2_^[^ ^51^ ^]^	0.064	2.4	1.2	—	5−0
abbreviated to CuP^[^ ^10^ ^]^	0.028	1.9	8.3	0.26	7−4
EuB_4_O_7_^[^ ^52^ ^]^	0.285	1.7	1.0	—	4−0
NaYbGeO_4_^[^ ^53^ ^]^	0.101	1.7	0.9	—	5−0
YbPt_2_Sn^[^ ^8^ ^]^	0.127	1.6	0.8	—	6−0
YbNi_4_Mg^[^ ^54^ ^]^	0.111	1.4	0.7	—	3−0
Er_2_Ti_2_O_7_^[^ ^11^ ^]^	0.148	1.3	2.2	0.53	7−1.5
NaGdP_2_O_7_^[^ ^55^ ^]^	0.209	1.2	1.0	—	5−0
EuCl_2_^[^ ^56^ ^]^	0.378	1.0	0.9	—	5−0
YbCu_4_Ni^[^ ^57^ ^]^	0.114	1.0	0.8	—	10−0
Gd_3_Ga_5_O_12_^[^ ^12^ ^]^	0.363	0.8	1.6	—	4−0
Na_2_SrCo(PO_4_)_2_^[^ ^58^ ^]^	0.059	0.8	2.0	—	6−0

### 2.2 Quasi‐Adiabatic Demagnetization Refrigeration

In the narrow range between the starting field *µ*_0_ *H*_sta_ ∼ 12 T and the end field *µ*_0_ *H*_end_ ∼ 11 T, *Γ* ^norm^ remains high across a range of temperatures 0.2–4.5 K (Figure 2a), strongly indicating GdZnPO's potential for adiabatic demagnetization refrigeration. Using heat switches (HSs), adiabatic demagnetization refrigeration cycles can be implemented as illustrated in Figure 1c,d. Several methods exist for implementing HSs;^[^ ^3^, ^4^, ^43^, ^44^ ^]^ in this work, we used the above setup (inset of Figure 2a) to perform the quasi‐adiabatic demagnetization refrigeration (Figure 3) due to the absence of HSs in our lab. By using a higher *H*_sta_, incorporating HSs, and employing a larger single‐crystal sample, the magnetic cooling performance of GdZnPO during each run (Figure 3) could be significantly improved under better adiabatic conditions. Since *τ* = *C*_tot_/*κ*_eff_, a larger single‐crystal sample leads to a greater *C*_tot_, a longer relaxation time *τ*, and thus better adiabatic conditions. Here, *κ*_eff_ represents the effective heat conductance between the sample and the heat sink.

**Figure 4:**
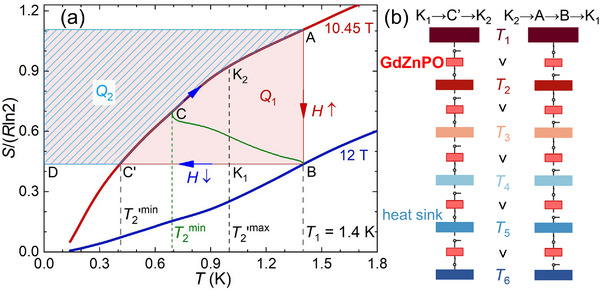
Magnetic refrigeration cycle. a) Entropy (*S*) per mole of GdZnPO, integrated from the specific heat (Figure S5, Supporting Information). The solid olive line shows the B→C route, *S*(*T*_2_, *H*), where *T*_2_ and *H* are measured by maintaining *T*_1_ = 1.4 K, as shown in Figure 3. b) Schematic blueprint of using multiple GdZnPO samples for magnetic cooling cycles.

When the applied field decreases from *H*_sta_ to *H*_end_, the GdZnPO spin system evolves from the polarized FM state to the degenerate SSL, absorbing heat due to the entropy increase during the quasi‐adiabatic demagnetization refrigeration and the subsequent constant‐*H*_end_ relaxation processes (B→C→A, see **Figure** **4**a). Figure 3 shows measurements during the processes B→C→A, with the heat sink temperature *T*_1_ maintained at various values from 0.2 to 4.5 K (dashed lines in Figure 3b). At *T*_1_ = 0.2 K, the lowest sample temperature *T*_2_^min^ = 36 mK is achieved by reducing the field from *µ*_0_ *H*_sta_ = 12 T to *µ*_0_ *H*_end_ = 11.2 T at a ramp rate of *v*_*H*_ = 0.3 Tmin^−1^, with good repeatability (Figure S2, Supporting Information). Our results demonstrate that efficient adiabatic demagnetization refrigeration using GdZnPO can be performed in a narrow field range between ∼11 and 12 T. Furthermore, the magnetic cooling performance of GdZnPO remains effective up to at least *T*_1_ = 4.5 K, above the temperature of liquid ^4^He (4.2 K) and well above T*. The normalized Grüneisen ratio is obtained as *Γ* ^norm^ ∼ (*H*/*T*_2_)(d*T*_2_/d*H*), and is roughly consistent with the high‐resolution value determined by the alternating‐field technique (Figure 2a). However, the Grüneisen ratio shown in the inset of Figure 3a was measured under non‐isothermal conditions with a large *T*_1_ − *T*_2_, making it less reliable. Here, *Γ* ^norm^ was underestimated due to the heat leak power, which is proportional to *T*_1_ − *T*_2_.

### 2.3 Magnetic Cooling Power and Efficiency

GdZnPO exhibits both a giant residual specific heat (*C*_0_ ∼ 1.2 JK^−1^mol^−1^)^[^ ^24^ ^]^ and a giant MCE (*Γ*_m_ ∼ 4.44 T^−1^) near *H*_c_, indicating high cooling power even at extremely low temperatures. The cooling power, *P*_C_ = *C*_tot_ *TΓ*_m_ *v*_*H*_, was evaluated at *T* = 0.2 K and *v*_*H*_ = 0.3 Tmin^−1^, yielding *P*_C_ ∼ 2.7, 5.0, 5.1, and 1.5 mWmol^−1^ at *µ*_0_ *H* = 10.8 (where *Γ*_m_ is low), 11, 11.5, and 11.8 T (where *C*_tot_ is small), respectively (Table S1, Supporting Information). This suggests that approximately 1/5 mol, or 50 g, or 8 cm^3^ of GdZnPO can generate a cooling power of ∼1 mW, comparable to the performance of a good ^3^He‐^4^He dilution refrigerator at low temperatures (∼0.2 K).

The entropy was integrated from the measured specific heat,^[^ ^24^ ^]^ with its reliability discussed in the Supporting Information. The magnetic refrigeration cycle of GdZnPO in the *S*‐*T* space at *T*_1_ = 1.4 K is shown in Figure 4a. In the isothermal process A→B, the applied field drives the system from the SSL to the FM state below T*, resulting in an entropy decrease and heat release, *Q*_1_ = *T*_1_(*S*_A_ − *S*_B_) (Figure 4a). The B→C′ process corresponds to an adiabatic demagnetization refrigeration process, with the GdZnPO spin system expected to reach its lowest temperature, $T_{2}^{\prime }$ ^min^. In our experiments, the system actually underwent a quasi‐adiabatic demagnetization refrigeration process that approximately followed B→C (solid olive line in Figure 4a), resulting in a slightly higher *T*_2_^min^. The C′→A process is a constant‐field relaxation process, with a decalescence contribution, *Q*_2_ = $\int_{C^{\prime }}^{A}TdS\left(T,H\equiv H_{\mathrm{end}}\right)$. We obtained a magnetic cooling efficiency of *Q*_2_/*Q*_1_ = 0.60(2) for GdZnPO at *T*_1_ = 0.7–1.8 K (Table S1, Supporting Information).

As shown in Figure 4a, the decalescence under actual quasi‐adiabatic conditions, $Q_{2}^{a}$ = $\int_{B}^{C}TdS+\int_{C}^{A}TdS$, is clearly larger than that under ideal adiabatic conditions, *Q*_2_ = $\int_{B}^{C^{\prime }}TdS+\int_{C^{\prime }}^{A}TdS$ = $\int_{C^{\prime }}^{A}TdS$, due to the additional area enclosed by path BC'CB. Consequently, the magnetic cooling efficiency under actual quasi‐adiabatic conditions, $Q_{2}^{a}$/*Q*_1_, exceeds the ideal adiabatic efficiency, *Q*_2_/*Q*_1_. This arises because the heat leakage increases the minimum achievable temperature, i.e., $T_{2}$ ^min^ > $T_{2}^{\prime }$ ^min^. In the specific case shown in Figure 4a for *T*_1_ = 1.4 K, we obtain *Q*_1_ = *T*_1_(*S*_A_ − *S*_B_) = 5.406 Jmol^−1^ per cycle, *Q*_2_ = 3.157 Jmol^−1^ per cycle, $T_{2}^{\prime }$ ^min^ = 0.409 K, $Q_{2}^{a}$ = 3.866 Jmol^−1^ per cycle, and $T_{2}$ ^min^ = 0.693 K. As a result, we find $Q_{2}^{a}$/*Q*_1_ (∼0.72) > *Q*_2_/*Q*_1_ (∼0.58) and $T_{2}$ ^min^ /*T*_1_ (∼0.50) > $T_{2}^{\prime }$ ^min^/*T*_1_ (∼0.29). Although the actual quasi‐adiabatic efficiency $Q_{2}^{a}$/*Q*_1_ can be higher than the ideal adiabatic efficiency *Q*_2_/*Q*_1_, the latter is independent of specific experimental conditions and thus better reflects the intrinsic cooling efficiency of a material.

The GdZnPO spin system exhibits no observable magnetic hysteresis down to at least 0.05 K (≪|*θ*_w_|, Figure S2, Supporting Information), consistent with the theoretical expectation of the classical SSL ansatz for the easy‐plane *J*_1_‐*J*_2_ honeycomb‐lattice model at zero temperature.^[^ ^27^ ^]^ Consequently, magnetic dissipation is negligible, and the system entropy is expected to be fully recoverable after a cooling cycle.

## 3 Discussion

As shown in Figure 2a, *Γ* ^norm^ or *Γ*_m_ peaks near the crossover field *µ*_0_ *H*_c_ ∼ 12 T, and remains high up to ∼4.5 K. Based on this, we propose using multiple GdZnPO samples for magnetic cooling cycles, directly cooling from a high temperature (∼4.5 K) down to ∼36 mK within the same superconducting magnet, as illustrated in Figure 4b. To ensure a temperature decrease from higher to lower heat sinks, when the GdZnPO sample temperature drops below a set value $T_{2}^{\prime }$ ^max^ (i.e., during K_1_→C′→K_2_, see Figure 4a), the HS between the higher sink and the sample should be turned off, and the HS between the sample and the lower sink should be turned on, as shown in Figure 1c. Conversely, when the sample temperature exceeds $T_{2}^{\prime }$ ^max^ (i.e., during K_2_→A→B→K_1_), the HS between the higher sink and the sample should be turned on, and the HS between the sample and the lower sink should be turned off, as shown in Figure 1d. To ensure a continuous temperature decrease from higher to lower sinks (i.e., *T*_*j* + 1_ < *T*_*j*_ for *j* = 1, 2, …, see Figure 4b), the HSs can be activated simultaneously at the K_1_ and K_2_ points during the adiabatic demagnetization refrigeration cycles, as shown in Figure 4.

While paramagnetic salts,^[^ ^59^ ^]^ spin supersolids,^[^ ^12^ ^]^ and heavy fermion metals^[^ ^54^, ^57^ ^]^ have been widely studied for magnetic cooling, the potential of SSLs remains largely unexplored. Our results demonstrate that the *s* = 7/2 honeycomb‐lattice SSL candidate GdZnPO offers a competitive advantage in magnetic cooling over other compounds (Table 1). The crossover field, *µ*_0_ *H*_c_ ∼ 12 T, arising from the strongly correlated magnetism of GdZnPO, is exceptionally high compared to other compounds. Consequently, efficient adiabatic demagnetization refrigeration cycles using GdZnPO can be performed above ∼11 T (see Figure 3), making it particularly useful when a high magnetic field is necessary. While this high operational field entails higher equipment costs, potentially limiting the practical application of GdZnPO in adiabatic demagnetization refrigeration, other refrigerants (like commercial Gd_3_Ga_5_O_12_^[^ ^12^, ^52^ ^]^) require high starting fields of *µ*_0_ *H*_sta_ ∼ 3‐10 T (Table 1), necessitating superconducting magnets. While increasing the superconducting magnet's maximum field to 12 T incurs additional costs, the energy consumption per cycle is roughly proportional to the operational field range, *µ*_0_(*H*_sta_−*H*_end_), which is lowest for GdZnPO (Table 1). The main superconducting coils can provide a steady ∼11 T field in persistent mode, requiring no energy input beyond cryogenic maintenance. The remaining ∼0‐1 T field can be cycled by secondary coils, as in commercial magnetic refrigerators. These features make GdZnPO a promising candidate for magnetic cooling applications.

Due to the extremely strong neutron absorption by Gd atoms, neutron scattering measurements on GdZnPO remain highly challenging. Nevertheless, several experimental observations support the emergence of a SSL in GdZnPO at low temperatures:

(1) Within the spherical approximation, the generic theory of SSLs predicts a distinctive low‐temperature behavior of the magnetic specific heat: *C*_m_ ∼ *C*_0_ + *C*_1_ *T*.^[^ ^14^, ^15^ ^]^ To our knowledge, this behavior has not been reported in other spin systems. In a 2D SSL, **Q**_G_ fluctuates along a continuous contour in reciprocal space, in stark contrast to conventional magnetic states where **Q**_G_ takes on discrete values. This fluctuation implies the existence of spin degrees of freedom along the spiral contour—analogous to the translational degrees of freedom in an ideal gas—which leads to a finite residual specific heat *C*_0_. *C*_0_ originates from zero‐energy excitations along the degenerate continuous contour in the classical limit (*s* → ∞), while the linear term *C*_1_ *T* arises from low‐energy excitations off the contour. To our knowledge, this low‐*T* behavior of *C*_m_ ∼ *C*_0_ + *C*_1_ *T* had not been experimentally observed in any compounds until our previous report on the SSL candidate GdZnPO, where this behavior appears below the crossover field *µ*_0_ *H*_c_ ∼ 12 T and down to ∼53 mK (≪|*θ*_w_|).^[^ ^24^ ^]^
(2) Within the quasi‐particle framework, the magnetic thermal conductivity of a SSL is expected to scale with the magnetic specific heat, i.e., $\kappa_{xx}^{m}$ ∼ *κ*_0_ + *κ*_1_ *T*, analogous to *C*_m_ ∼ *C*_0_ + *C*_1_ *T*. Recently, we observed a large thermal conductivity in GdZnPO that closely follows the expected form *κ*_*xx*_ = *κ*_0_ + *κ*_1_ *T* + *K*_p_ *T* ^3^ below 1 K and up to the crossover field *µ*_0_ *H*_c_ ∼ 12 T, where the *K*_p_ *T* ^3^ term arises from phonons.^[^ ^60^ ^]^ Notably, the observation of a magnetic contribution $\kappa_{xx}^{m}$ ∼ *κ*_0_ + *κ*_1_ *T* is highly distinctive and, to our knowledge, has not been reported in any other magnetic compound.
(3) Low‐temperature magnetization measurements clearly reveal easy‐plane anisotropy in GdZnPO.^[^ ^24^ ^]^ Taking into account the crystal structure and magnetization data, the spin system is well described by the easy‐plane *J*_1_‐*J*_2_ honeycomb‐lattice model.^[^ ^24^ ^]^ Theoretically, this model stabilizes a SSL over a broad parameter range, ∣*J*_2_/*J*_1_∣ > 1/6, and the SSL persists down to very low temperatures.^[^ ^30^ ^]^ Moreover, the experimentally determined Hamiltonian yields a crossover field of *µ*_0_ *H*_c_ = $s\left[2D+3J_{1}+9J_{2}+J_{1}^{2}/\left(4J_{2}\right)\right]/\left(g\mu_{B}\right)$ ∼ 12 T and a Curie‐Weiss temperature of *θ*_w_ = −*s*(*s* + 1)(*J*_1_ + 2*J*_2_) ∼ −12 K, both of which are in good agreement with experimental observations on GdZnPO (see Figure 2).^[^ ^24^ ^]^
(4) Within the SSL ansatz on the honeycomb lattice,^[^ ^27^ ^]^ the zero‐temperature magnetic susceptibility is theoretically predicted to remain constant up to *µ*_0_ *H*_c_. Specifically, the calculated parallel susceptibility is given by $\chi_{\mathrm{cal}}^{∥}$ = $\mu_{0}N_{A}g^{2}\mu_{B}^{2}/\left[2D+3J_{1}+9J_{2}+J_{1}^{2}/\left(4J_{2}\right)\right]$+$\chi_{\mathrm{vv}}^{∥}$, which yields a value of ∼4.4 cm^3^mol^−1^ for GdZnPO, in good agreement with experimental results down to 50 mK (∼0.4%∣*θ*_w_∣, Figure S3, Supporting Information).^[^ ^24^ ^]^
(5) In this work, the giant magnetocaloric effect observed in GdZnPO is well captured by the experimentally determined easy‐plane *J*_1_‐*J*_2_ honeycomb‐lattice spin Hamiltonian,^[^ ^24^ ^]^ without the need for adjustable microscopic interaction parameters (see Figure 2)—further supporting the presence of a SSL.

Interlayer couplings can effectively increase the system's dimensionality from two to three. Both this dimensional crossover and the spatial anisotropy of the honeycomb lattice tend to relieve magnetic frustration, thereby destabilizing the SSL at low temperatures. For example, the honeycomb‐lattice compound FeCl_3_ exhibits a clear instability of the SSL at ∼5 K, likely due to significant interlayer interactions.^[^ ^39^ ^]^ In contrast, interlayer couplings in GdZnPO are negligible, owing to the highly localized nature of the Gd^3+^ 4*f* electrons and the large interlayer spacing of *c*/3 ∼ 10.2 Å, where magnetic layers are separated by nonmagnetic ZnP bilayers.^[^ ^24^ ^]^ Furthermore, the spatial isotropy of the honeycomb lattice in GdZnPO is protected by the $R\bar{3}m$ space group symmetry. As a result, measurements on GdZnPO show no clear indication of instability from the SSL into a conventionally ordered state below *µ*_0_ *H*_c_, down to at least ∼50 mK (∼0.4%∣*θ*_w_∣).

In the frustrated honeycomb‐lattice antiferromagnet GdZnPO, the field‐induced crossover from the non‐degenerate FM state (*H* ⩾ *H*_c_) to the putative degenerate SSL (*H* < *H*_c_) leads to a significant increase in entropy, decalescence, and a giant MCE, near the crossover field *H*_c_ at low temperatures. We demonstrate that GdZnPO is an efficient material for magnetic cooling, operating effectively within a narrow field range (∼11–12 T, near *µ*_0_ *H*_c_), with high cooling power and excellent performance across a wide temperature range (∼0.036–4.5 K). Our findings not only highlight the potential of GdZnPO for magnetic cooling applications but also open avenues for further research into other SSL candidates for similar purposes.

## 4 Experimental Section

### Samples

Single crystals of GdZnPO were prepared using the two‐step method.^[^ ^24^ ^]^ The resulting crystals exhibit generally high quality, as evidenced by sharp and clear Laue diffraction patterns (Figure S3, Supporting Information), and no significant sample dependence of thermodynamic properties has been observed in the recent studies.^[^ ^60^ ^]^ In general, finite‐size effects on thermodynamic properties become negligible when the system exceeds ∼1000 spins along each dimension. Consequently, macroscopic structural defects such as grain boundaries have minimal impact on thermodynamic measurements. Therefore, the magnetocaloric effect (*Γ*_m_ = −(d*M*/d*T*)/*C*_p_) and the associated magnetic cooling performance—both governed by thermodynamic properties—are expected to be largely insensitive to structural imperfections in the case of GdZnPO.

### Experimental Methods

Twelve *ab*‐plane single crystals (total mass 5.37 mg), aligned with the *c* axis along the applied magnetic field, were used to measure MCE and conduct quasi‐adiabatic demagnetization refrigeration in a ^3^He–^4^He dilution refrigerator. As shown in the inset of Figure 2a, a weak thermal link between the sample and the Cu heat sink was established using a thin constantan wire. The MCE was measured using the high‐resolution alternating‐field technique under quasi‐adiabatic conditions,^[^ ^13^, ^40^, ^61^ ^]^ with a frequency of *ν* = 0.005 or 0.01 Hz. Specific heat and magnetization of GdZnPO at *T* = 0.05–1.8 K and *µ*_0_ *H* = 0‐12 T were measured in the dilution refrigerator.^[^ ^24^, ^62^, ^63^, ^64^ ^]^ Both the MCE and the quasi‐adiabatic demagnetization refrigeration results are highly reproducible (Figure S2, Supporting Information). Further details on the experimental procedures are provided in the Supporting Information.

### Numerical Methods

Standard MC simulations of the thermodynamic quantities (Figure 2b) were performed on a 2× 72^2^ cluster with periodic boundary conditions, using the previously determined Hamiltonian of GdZnPO without parameter tuning.^[^ ^24^ ^]^ 5000 MC steps were conducted at each of 200 temperatures, gradually annealing from 50 K to 0.05 K, with 1000 steps allocated for thermalization. The calculated quantities were averaged over four independent samples. The MC algorithm relies on repeated random sampling, which inherently introduces noise in the calculated *Γ*_m_, especially at low temperatures. This noise is random in nature rather than oscillatory, as its pattern varies noticeably at a different temperature (see Figure 2b).

### Statistical Analysis

All statistical analyses were performed using OriginPro 2018.

## Conflict of Interest

The authors declare no conflict of interest.

## Supporting information

Supporting Information

## Data Availability

The data that support the findings of this study are available from the corresponding author upon reasonable request.
