# Supplementary material for: Giant Magnetocaloric Effect in a Honeycomb Spiral Spin‐Liquid Candidate
Source: Adv Sci (Weinh). 2025 Aug 26;12(43):e10086. doi: 10.1002/advs.202510086 (PMC12631906; doi:10.1002/advs.202510086)
Supplement: Supplementary file 1 — Supporting Information [file ADVS-12-e10086-s001.pdf]

Supporting Information for "Giant Magnetocaloric Effect in a Honeycomb Spiral Spin-Liquid Candidate"

*Yuqian Zhao, Xun Chen, Zongtang Wan, Zhaohua Ma, and Yuesheng Li**

Wuhan National High Magnetic Field Center and School of Physics, Huazhong University of Science and Technology, 430074 Wuhan, China
Email Address: *yuesheng_li@hust.edu.cn

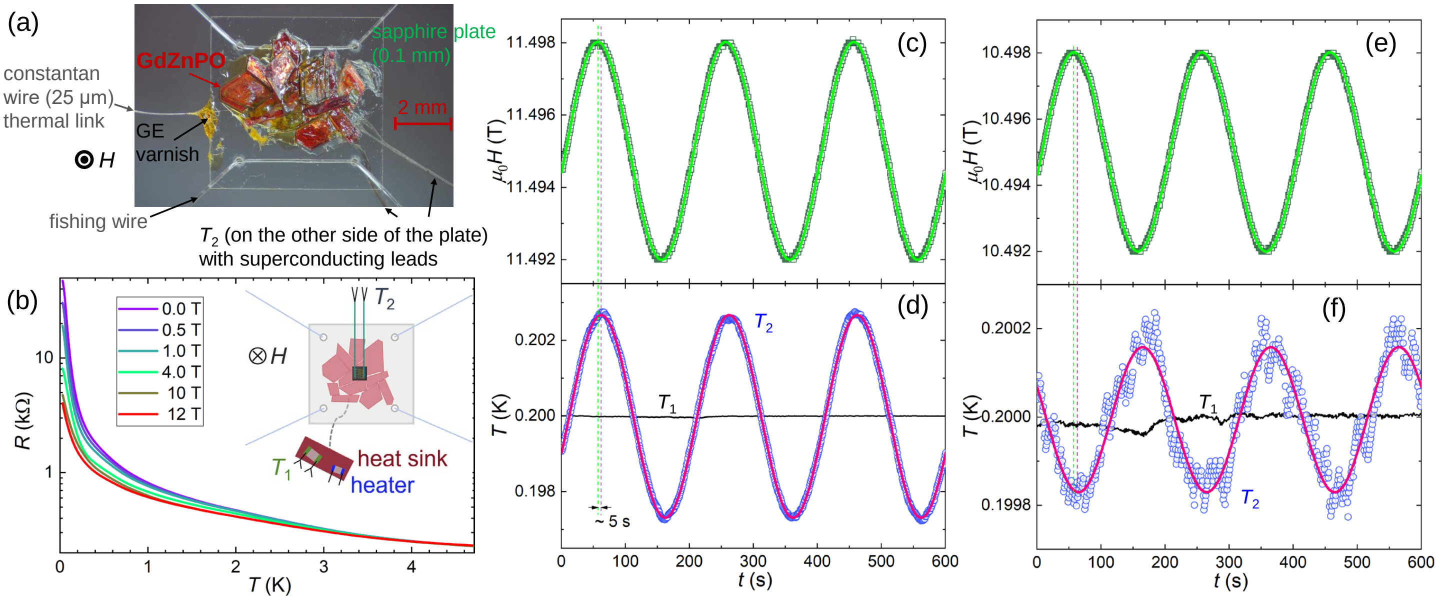

Figure S1: Magnetocaloric effect measured on GdZnPO. a) Single-crystal sample of GdZnPO used in the magnetocaloric and quasi-adiabatic demagnetization refrigeration measurements, viewed from above. b) Temperature and magnetic field dependence of the resistance of thermometer T_2 (CX-1010-BR, Lakeshore). Thermometer T_2 was *in situ* calibrated against the reference thermometer T_1 (RX-102B-RS-0.02B, Lakeshore, calibrated between 0.018 and 45 K). The inset shows a diagrammatic sketch of the experimental setup, viewed from below. c) and e) Time (t) dependence of the applied magnetic field, and d) and f) corresponding temporal variations in temperatures T_2 and T_1. The colored lines represent the fits to the sine wave signals, $\mu_0\Delta H\sin[2\pi\nu(t-t_H)]+\mu_0H^{\rm av}$ and $\Delta T\sin[2\pi\nu(t-t_T)]+T^{\rm av}$. The raw T_2 data were fitted using $\Delta T\sin[2\pi\nu(t-t_T)]+T^{\rm av}+a_1[t-\max(t)]+a_2[t-\max(t)]^2$, similar to the approach in Ref. [1]. To clarify the sine wave signal of T_2, we display $T_2-a_1[t-\max(t)]-a_2[t-\max(t)]^2$ in d) and f). The heat sink temperature, T_1, was maintained at 0.2 K using the heater shown in the inset of b).

1 Magnetocaloric effect and quasi-adiabatic demagnetization refrigeration

Single crystals of GdZnPO were grown as described in Ref. [2]. The crystals are planar, transparent, reddish-brown insulators, with the c axis perpendicular to the plane, as confirmed by x-ray diffraction [2]. For magnetocaloric and quasi-adiabatic demagnetization refrigeration measurements, 12 high-quality single crystals with a total mass of 5.37 mg were selected (Figure S1a). The crystals were mounted on the upper surface of a sapphire plate (thickness ~0.1 mm) using moderate GE varnish, with the plate suspended by a thin fishing wire. A thermometer T_2 (CX-1010-BR, Lakeshore) with superconducting leads was attached to the lower surface of the sapphire plate, and the plate was thermally linked to a Cu heat sink via a constantan wire (25 μm diameter, ~5 cm length) (see inset of Figure S1b). The heat sink temperature was monitored with a reference thermometer T_1 (RX-102B-RS-0.02B, Lakeshore, calibrated between 0.018

and 45 K) and regulated using a chip resistor heater. The sample thermometer T_2 was *in situ* calibrated against the reference thermometer T_1, at T = 0.03-4.7 K and $\mu_0 H$ = 0-12 T (Figure S1b). Temperature measurements for both T_1 and T_2, as well as T_1 maintenance, were carried out using an ac resistance Bridge & temperature controller (model 372, Lakeshore) with internal lock-in amplifiers. The alternating magnetic field (frequency ν = 0.005 or 0.01 Hz, amplitude $\mu_0 \Delta H$ = 2 or 3 mT, and average field $\mu_0 H^{\rm av}$ = 0-12 T) was generated by a superconducting magnet (INTA-LLD-S12/14, Oxford Instruments). The heat sink temperature T_1 was lowered to 0.03 K using a ^{3}He-^{4}He dilution refrigerator (KELMX-400, Oxford Instruments), while measurements at $T_1 \geq 3$ K were performed with the dilution refrigerator turned off.

The alternating magnetic field of $\mu_0 \Delta H \sin[2\pi\nu(t-t_H)] + \mu_0 H^{\rm av}$ induces a sine wave signal for T_2, given by $T_2 \sim \Delta T \sin[2\pi\nu(t-t_T)] + T^{\rm av}$, as shown in Figure S1. At ν = 0.005 Hz, the fits yield a small delay $\nu(t_T - t_H) \sim 0.025$ ($\ll 1$), indicating good thermal contact between the sample and the thermometer T_2 [1]. Under quasi-adiabatic conditions ($\tau^{-1} \ll 2\pi\nu$), the magnetic Grüneisen parameter is determined by the alternating-field technique as $\mathit{\Gamma}_{\rm m}$ ($T = T^{\rm av} \sim T_1$, $H = H^{\rm av}$) = $\Delta T/(T\mu_0\Delta H)$ (Figure S2a). Here, τ = 400-600 s is the relaxation time, as listed in Figure S2c. For instance, at T = 0.2 K and $\mu_0 H$ = 11.495 T, the fits yield $\mu_0 \Delta H \sim 0.003$ T and $\Delta T \sim 0.00267$ K (see Figure S1c,d), resulting in $\mathit{\Gamma}_{\rm m} \sim 4.44$ T^{-1} and $\mathit{\Gamma}^{\rm norm} = \mu_0 H \mathit{\Gamma}_{\rm m} \sim 51$ (see main text). Doubling the frequency to 0.01 Hz does not significantly affect $\mathit{\Gamma}_{\rm m}$ (see Figure S2a), confirming the quasi-adiabatic conditions of the experiments. Additionally, $\mathit{\Gamma}_{\rm m}$ is nearly independent of the excitation amplitude ΔH, indicating a good linear response.

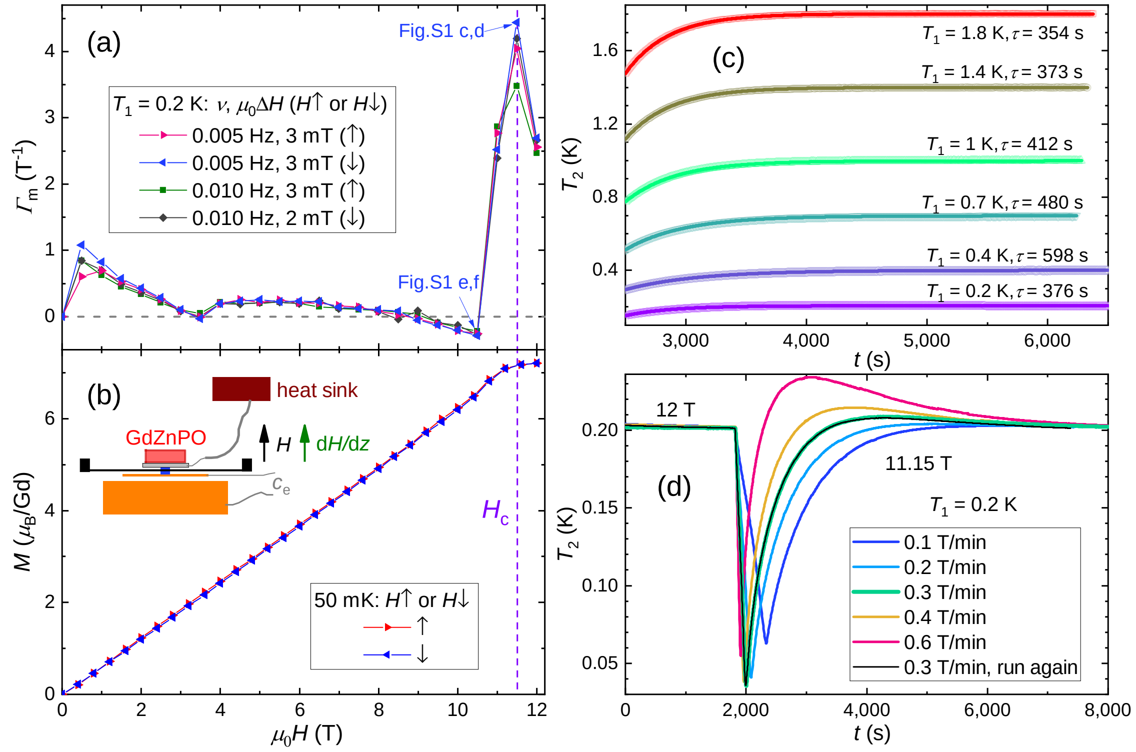

Figure S2: Thermodynamic properties of GdZnPO and magnetic cooling, with the magnetic field applied along the c axis ($H \parallel c$). a) Magnetic Grüneisen parameter measured by maintaining T_1 = 0.2 K. b) Magnetization measured at 50 mK. The inset shows a diagrammatic sketch of the capacitive Faraday magnetometer. c) Time (t) dependence of T_2 during constant-field relaxation processes at various T_1. The colored lines represent the relaxation fits to the experimental data for $t >$ 2,500 s, T_2 = $(T_2^0 - T_2^\infty)\exp(-t/\tau) + T_2^\infty$, where the fitted relaxation times τ are listed. d) Quasi-adiabatic demagnetization refrigeration using GdZnPO at various ramp rates, with the field varying from $\mu_0 H_{\rm sta}$ = 12 T to $\mu_0 H_{\rm end}$ = 11.15 T, while maintaining T_1 = 0.2 K.

Maintaining T_1 = 0.2 K, the quasi-adiabatic demagnetization refrigeration in our setup exhibits a clear dependence on the ramp rate v_H from the starting field $H_{\rm sta}$ to the end field $H_{\rm end}$, as shown in Figure S2d. The lowest achievable temperature is $T_2^{\rm min} \sim 36$ mK at $v_H \sim 0.3$ T/min, which is highly reproducible.

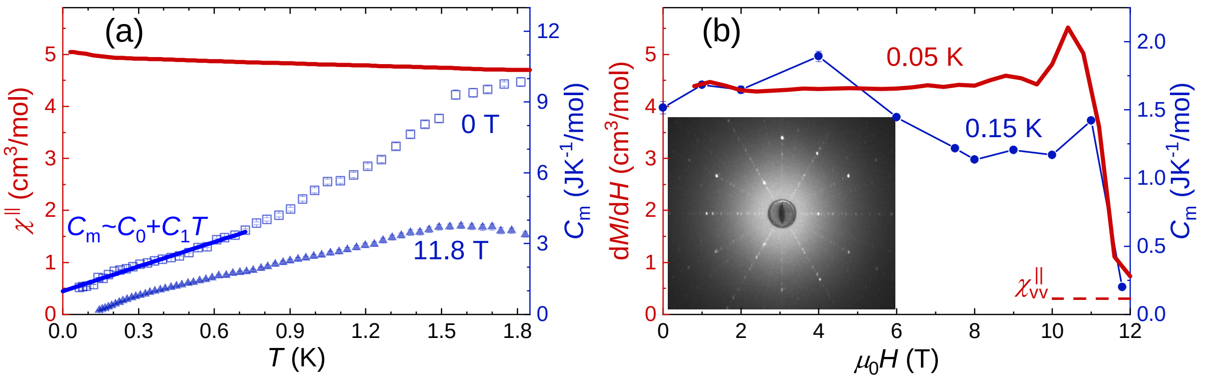

Figure S3: Thermodynamic properties of GdZnPO. a) Temperature dependence of the magnetic susceptibility (measured along the c axis) and specific heat. The blue line represents a linear fit to the zero-field specific heat data below 0.7 K, following $C_{\mathrm{m}} \sim C_0 + C_1 T$. b) Magnetic field dependence of the susceptibility (dM/dH, at 0.05 K) and specific heat (at 0.15 K). The dashed line indicates the Van Vleck susceptibility, $\chi_{\mathrm{vv}}^{\parallel} \sim 0.3$ cm^3/mol [2]. The inset shows the Laue x-ray diffraction pattern of the ab-plane for a representative single crystal of GdZnPO.

The cooling power, $P_{\mathrm{C}} = C_{\mathrm{tot}} T_2 \mathit{\Gamma}_{\mathrm{m}} v_H$, is proportional to the ramp rate v_H. When v_H or T_2 is too low, P_{C} can become comparable to the heat leak power, $P_{\mathrm{L}} = C_{\mathrm{tot}}(T_1 - T_2)/\tau$, which severely limits the magnetic cooling performance. On the other hand, if v_H is too high, significant eddy heat may be generated in metal parts at low temperatures (~0.2 K). Therefore, we primarily used $v_H = 0.3$ T/min for $T_1 \leq 1.8$ K, and v_H = 0.6 T/min for $T_1 \geq 3$ K (see main text).

2 Magnetization

The magnetization (M) of GdZnPO (total mass 13.1 mg) was measured under a magnetic field of $\mu_0 H$ = 0-12 T applied along the c axis using a high-resolution capacitive Faraday magnetometer (see inset of Figure S2b) at temperatures between 0.05 K and 1.8 K [3] in the same dilution refrigerator. A strong thermal link between the single-crystal sample and the heat sink was established using an Ag foil (~4 μm × 2 mm × 10 mm), an Ag line (diameter 0.2 mm, length ~10 cm), and silver paste to minimize the temperature difference. In this study, $M(T, H)$ was determined in steady-temperature and steady-field mode. At each temperature and magnetic field, the electrical capacitance was recorded once it became nearly time-independent. The Faraday force was induced by a magnetic field gradient, $F = M\mu_0 \mathrm{d}H/\mathrm{d}z$, where $\mu_0 \mathrm{d}H/\mathrm{d}z = \pm 3$ T/m was generated by the gradient superconducting coils in the magnet [4]. The electrical capacitance c_{e} of the magnetometer was measured using a digital capacitance bridge (AH-2550A, Andeen-Hagerling, Inc.) with the three-terminal method. The parallel loss (~0.037 nS) was negligible, and the parallel capacitance (~3.7 pF) provided an accurate measure of c_{e} [2]. The magnetization was then obtained as $M = f_{\mathrm{eff}}[c_{\mathrm{e}}(\mu_0 \mathrm{d}H/\mathrm{d}z = +3\ \mathrm{T/m})^{-1} - c_{\mathrm{e}}(\mu_0 \mathrm{d}H/\mathrm{d}z = -3\ \mathrm{T/m})^{-1}]$, where the prefactor f_{eff} = 1670 pF·μ_{B}/Gd was determined by scaling to the magnetization measured using a magnetic property measurement system (Quantum Design) at 1.8 K [4].

As shown in Figure S2b, the low-temperature magnetization is proportional to the applied magnetic field up to ~10.5 T, well consistent with the spiral spin liquid (SSL) ground-state ansatz [2]. At $\mu_0 H \sim$ 10.5-11.6 T, the high-resolution magnetization data clearly exhibit a hump (Figure S2b), indicating a field-induced critical behavior. At $\mu_0 H \sim 11.5$ T, the magnetic Grüneisen parameter $\mathit{\Gamma}_{\mathrm{m}}$ peaks (Figure S2a) and the magnetization M approaches its fully polarized value. A weak Van Vleck magnetization, $M_{\mathrm{vv}} = \chi_{\mathrm{vv}} H$ (~0.4 μ_{B}/Gd), where $\chi_{\mathrm{vv}} \sim 0.3$ cm^3/mol [2], should also contribute to M at $\mu_0 H \sim 12$ T. At low temperatures ($T < T^* \sim 2$ K), the GdZnPO spin system stabilizes in the putative SSL for $H < H_{\mathrm{c}}$ but becomes substantially polarized for $H \geq H_{\mathrm{c}}$, where $\mu_0 H_{\mathrm{c}} \sim 11.5$ T (~12 T) is the crossover field. Moreover, no evident magnetic hysteresis is observed during the field sweep in M measurements (see Figsure S2b),

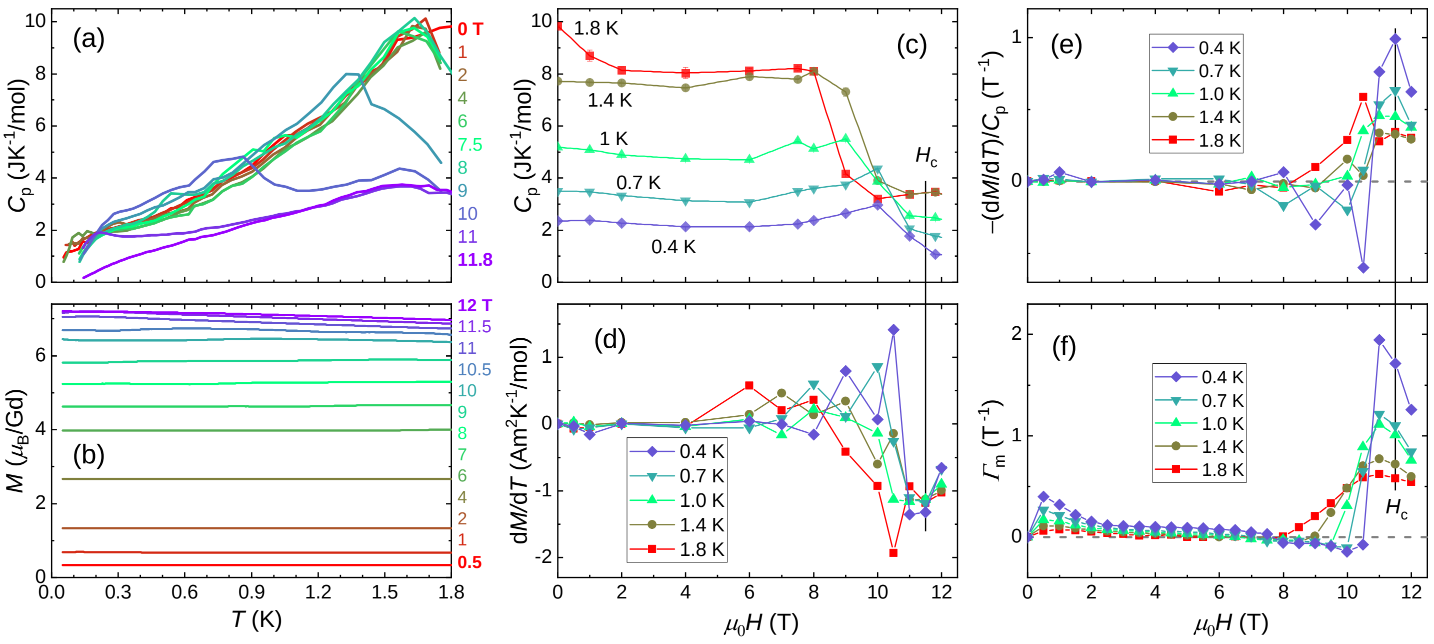

Figure S4: Magnetic Grüneisen parameter indirectly obtained from other thermodynamic quantities. a,b) Temperature dependence of specific heat (C_p) and magnetization (M) measured on GdZnPO single crystals, under various magnetic fields applied along the c axis ($H \parallel c$). c,d) Magnetic field dependence of C_p and $\mathrm{d}M/\mathrm{d}T$ derived from panels a) and b), respectively. Comparison of $-(\mathrm{d}M/\mathrm{d}T)/C_\mathrm{p}$ e) with the magnetic Grüneisen parameter $\mathit{\Gamma}_\mathrm{m}$ f) as directly determined by the high-resolution alternating-field technique.

suggesting that the field-induced crossover is second-order or higher.

The M-H data measured at 0.05 K ($\ll |\theta_\mathrm{w}|$, where $\theta_\mathrm{w} = -s(s+1)(J_1 + 2J_2) = -12$ K is the Curie-Weiss temperature) is shown in Figsure S2b, and its first derivative ($\mathrm{d}M/\mathrm{d}H$) is displayed in Figsure S3b. Below the crossover field $\mu_0 H_\mathrm{c} \sim 12$ T, $\mathrm{d}M/\mathrm{d}H \sim \mu_0 N_\mathrm{A} g^2 \mu_\mathrm{B}^2/[2D + 3J_1 + 9J_2 + J_1^2/(4J_2)] + \chi_\mathrm{vv}^{\parallel} \sim 4.4$ cm^3/mol, consistent with the classical ($s \to \infty$) SSL ansatz on the honeycomb lattice [5]. Above $\mu_0 H_\mathrm{c}$, the classical spin model becomes fully polarized, as shown in Fig. 4(a) of our previous work [2]. No intermediate phase between the SSL and polarized state is expected in the classical easy-plane frustrated honeycomb-lattice model [5]. Interestingly, the low-T $\mathrm{d}M/\mathrm{d}H$ data for GdZnPO reveals a weak peak near 10.5 T (see Figsure S3b), which deviates slightly from classical predictions. This discrepancy is most likely due to quantum fluctuations associated with the finite spin quantum number $s = 7/2$ of Gd^{3+} ions. This quantization reduces the total entropy per site from an infinite value (in the classical limit) to $R\ln(2s+1) = 3R\ln 2$. However, theoretical and numerical studies of the easy-plane J_1-J_2 honeycomb-lattice model have thus far been limited to the classical limit, and accurate simulations of a frustrated $s = 7/2$ quantum spin system remain extremely challenging. Therefore, based on the available M-H data, there is currently no strong evidence for the existence of intermediate phases between the putative SSL and the polarized state.

3 Magnetic cooling cycle

Detailed low-temperature specific heat measurements on GdZnPO were reported in our previous work [2]. Below 1.8 K, the lattice contribution, C_lat, as estimated from measurements on the nonmagnetic reference compound YZnPO, is negligible compared to the total specific heat C_p of GdZnPO, $C_\mathrm{lat} \lesssim 0.002 C_\mathrm{p}$. Therefore, the lattice contribution can be safely neglected in this study below 1.8 K. Figure S4a shows the specific heat measured at various fields, ranging from 0 to 11.8 T, applied along the c axis, with some data previously reported in Ref. [2].

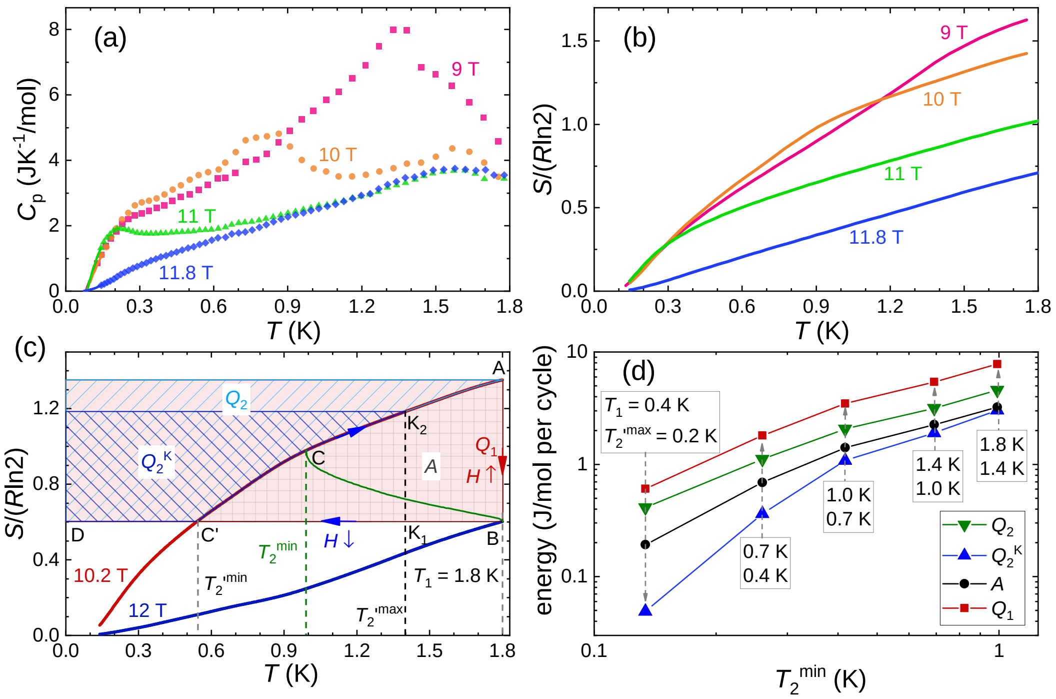

Figure S5: Magnetic entropy and cooling efficiency. a) Temperature dependence of specific heat (C_p) measured at $\mu_0 H = 9$, 10, 11, and 11.8 T. The colored lines represent T-polynomial extrapolations of the experimental data down to temperatures (T_0) where $C_\mathrm{p} \sim 0$. b) Entropy of GdZnPO, $S(T) = \int_{T_0(C_\mathrm{p}\sim 0)}^{T} [C_\mathrm{p}(T')/T']\mathrm{d}T'$. c) Interpolation and extrapolation of $S(T,H)$ at $\mu_0 H = 10.2$ and 12 T, respectively. The solid olive line shows the quasi-adiabatic demagnetization refrigeration route B→C, $S(T_2, H)$, where T_2 and H are measured by maintaining $T_1 = 1.8$ K (see main text). The characteristic temperatures $T_2'^{\mathrm{min}}$, T_2^{min}, $T_2'^{\mathrm{max}}$ (set at 1.4 K), and T_1 are indicated. d) Energies per mole of GdZnPO during an adiabatic demagnetization refrigeration cycle. The corresponding set of T_1 and $T_2'^{\mathrm{max}}$ values are listed.

The measured $\mathit{\Gamma}_\mathrm{m}$ (Figure S4f) and C_p (Figure S4c) suggest that the maximum value of $|\mathrm{d}M/\mathrm{d}T|$ ($\sim|\mathit{\Gamma}_\mathrm{m} C_\mathrm{p}|$) is small, $\sim$2 Am^{2}K^{-1}/mol ($\sim$0.4 μ_BK^{-1}/Gd), comparable to the resolution of the capacitive Faraday magnetometer. As a result, the T-derivative data ($\mathrm{d}M/\mathrm{d}T$) remain noisy, as shown in Figure S4d, despite re-measurements of magnetization (M) at steady-T and -H mode (see Figure S4b). However, at low temperatures (below $\sim$0.6 K), M increases at $\sim$12 T (with $\mathrm{d}M/\mathrm{d}T < 0$) and decreases at $\sim$10.5 T (with $\mathrm{d}M/\mathrm{d}T > 0$) as the temperature is lowered, roughly consistent with classical Monte Carlo simulations (see main text). Moreover, $-(\mathrm{d}M/\mathrm{d}T)/C_\mathrm{p}$ (Figure S4e) shows agreement with the high-resolution Grüneisen parameter, $\mathit{\Gamma}_\mathrm{m}$, determined by the alternating-field technique (Figure S4f).

Figure S5a shows the specific heat of GdZnPO measured at $\mu_0 H = 9$, 10, 11, and 11.8 T. In the absence of experimental data below $\sim$0.15 K, we extrapolated the data using T-polynomials down to temperatures (T_0) where $C_\mathrm{p} \sim 0$, and then obtained the entropy using $S(T) = \int_{T_0(C_\mathrm{p}\sim 0)}^{T} [C_\mathrm{p}(T')/T']\mathrm{d}T'$ (see Figure S5b). Additionally, we interpolated and extrapolated the entropy $S(T,\,H)$ at $\mu_0 H = 9$, 10, 11, and 11.8 T to obtain $S(T,\,H)$ at other fields between 9 and 12 T (see Figure S5c and main text). At high temperatures ($\gg$ 0.15 K), the obtained entropy is sufficiently accurate, though its reliability decreases at lower temperatures (see below).

Figure S5c illustrates the adiabatic demagnetization refrigeration cycle using GdZnPO in the S-T space at $T_1 = 1.8$ K. In the isothermal process A→B, the applied field drives the spin system from the SSL to the substantially polarized ferromagnetic phase, leading to a decrease in entropy and a heat release $Q_1 = T_1(S_\mathrm{A} - S_\mathrm{B})$ ($\sim$7.786 J/mol). The process B→C′ is adiabatic demagnetization, and the spin system is expected to reach the lowest temperature $T_2'^{\mathrm{min}} \sim 0.541$ K. The C′→A process is a constant-field relaxation, with a decalescence heat content $Q_2 = \int_{\mathrm{C}'}^{\mathrm{A}} T\mathrm{d}S(T, H \equiv H_\mathrm{end})$ ($\sim$4.572 J/mol). As a result, the magnetic cooling efficiency is $Q_2/Q_1 \sim 0.587$, and the magnetic field work per adiabatic demagnetization refrigeration

cycle, $A = Q_1 - Q_2$ (~3.214 J/mol), is obtained.

In our experiments, the GdZnPO spin system underwent a quasi-adiabatic demagnetization refrigeration process, approximately following the B→C path (solid olive line in Figure S5c). The sample temperature T and magnetic field H were monitored in real-time, allowing us to track the quasi-adiabatic demagnetization refrigeration process in the S-T space. The spin system reached its lowest temperature, ${T_2}^{\rm min} \sim 0.992$ K, at the end of the quasi-adiabatic demagnetization refrigeration process, for $T_1 = 1.8$ K.

To maintain a lower temperature for the lower heat sink (see main text), where $T_2 < T_2'^{\rm max}$, the heat switch (HS) between the higher sink and the GdZnPO sample should be turned on, while the HS between the GdZnPO sample and the lower sink should be turned off, during the processes $\rm K_2 \rightarrow A \rightarrow B \rightarrow K_1$. Conversely, in the processes $\rm K_1 \rightarrow C' \rightarrow K_2$, the HS between the higher sink and the sample should be turned off, and the HS between the sample and the lower sink should be turned on, as shown in Figure S5c and Figure 1 of main text . When $T_2'^{\rm max}$ is set at 1.4 K, the heat absorbed from the lower sink was estimated as $Q_2^{\rm K} = \int_{\rm C'}^{\rm K_2} T {\rm d}S(T, H \equiv H_{\rm end})$ (~3.037 J/mol) at $T_1 = 1.8$ K. The energies Q_2, $Q_2^{\rm K}$, A, and Q_1 at various heat sink temperatures T_1 are summarized in Table S1 and Figure S5d. At $T_1 = 0.4$ K, the expected $T_2'^{\rm min}$ ~ 0.164 K after adiabatic demagnetization refrigeration, estimated from the entropy, is even larger than the experimental value of $T_2'^{\rm min} \sim 0.134$ K under quasi-adiabatic conditions, which may stem from the imprecise extrapolation of specific heat data at low temperatures (Figure S5a).

Table S1: Magnetic cooling efficiency and power of single-crystal GdZnPO, with the magnetic field applied along the c axis. The cooling power, $P_{\rm C} = C_{\rm p} T_1 \mathit{\Gamma}_{\rm m} v_H$, was obtained at 10.8, 11, 11.5, and 11.8 T, using the experimental specific heat $C_{\rm p}(T_1)$, magnetic Grüneisen parameter $\mathit{\Gamma}_{\rm m}(T_1)$, and a ramp rate of $v_H = 0.3$ T/min.

T_1 (K)	1.8	1.4	1.0	0.7	0.4	0.2
starting field $\mu_0 H_{\rm sta}$ (T)	12	12	12	12	12	12
end field $\mu_0 H_{\rm end}$ (T)	10.2	10.45	10.68	10.85	11.02	11.15
${T_2}^{\rm min}$ (K)	0.992	0.693	0.417	0.260	0.134	0.036
$T_2'^{\rm min}$ (K)	0.541	0.409	0.276	0.220	0.164	-
$T_2'^{\rm max}$ (K)	1.4	1	0.7	0.4	0.2	-
${T_2}^{\rm min}/T_1$	0.551	0.495	0.417	0.371	0.335	0.180
Q_2 (J/mol per cycle)	4.572	3.157	2.062	1.112	0.412	-
$Q_2^{\rm K}$ (J/mol per cycle)	3.037	1.910	1.082	0.363	0.049	-
A (J/mol per cycle)	3.214	2.249	1.408	0.689	0.193	-
Q_1 (J/mol per cycle)	7.786	5.406	3.469	1.801	0.605	-
Q_2/Q_1	0.587	0.584	0.594	0.618	0.681	-
$P_{\rm C}$(10.8 T) (mW/mol)	18.7	17.8	14.6	9.4	4.9	2.7
$P_{\rm C}$(11 T) (mW/mol)	19.2	18.1	14.3	8.8	6.9	5.0
$P_{\rm C}$(11.5 T) (mW/mol)	18.5	17.5	12.0	5.9	4.2	5.1
$P_{\rm C}$(11.8 T) (mW/mol)	17.5	15.9	10.6	5.7	2.6	1.5

References

[1] Y. Tokiwa, P. Gegenwart, *Rev. Sci. Instrum.* **2011**, *82* 013905.

[2] Z. Wan, Y. Zhao, X. Chen, Z. Ma, Z. Li, Z. Ouyang, Y. Li, *Phys. Rev. Lett.* **2024**, *133* 236704.

[3] Y. Shimizu, Y. Kono, T. Sugiyama, S. Kittaka, Y. Shimura, A. Miyake, D. Aoki, T. Sakakibara, *Rev. Sci. Instrum.* **2021**, *92* 123908.

[4] Y. Zhao, Z. Ma, Z. He, H. Liao, Y.-C. Wang, J. Wang, Y. Li, *Nat. Commun.* **2024**, *15* 3495.

[5] K. Fujiwara, S. Kitamura, T. Morimoto, *Phys. Rev. B* **2022**, *106* 035113.
